# Evaluation of SMA-13 Asphalt Mixture Reinforced by Different Types of Fiber Additives

**DOI:** 10.3390/ma17225468

**Published:** 2024-11-08

**Authors:** Haochen Wu, Peng Xiao, Ziyun Fei, Aihong Kang, Xing Wu

**Affiliations:** 1 College of Architectural Science and Engineering, Yangzhou University, Yangzhou 225100, China; 17715878456@163.com (H.W.); 13961740804@163.com (Z.F.); ahkang@yzu.edu.cn (A.K.); 2 Department of Architecture Built Environment and Construction Engineering, Politecnico di Milano, Piazza Leonardo da Vinci, 32, 20133 Milan, Italy; xing.wu@polimi.it

**Keywords:** fiber additives, SMA-13 mixture, mechanical property, dynamic mechanical response, basalt fiber, lignin fiber

## Abstract

This research aims at systematically evaluating the properties of SMA-13 asphalt mixture reinforced by several fiber additives including flocculent lignin fiber (FLF), granular lignin fiber (GLF), chopped basalt fiber (CBF), and flocculent basalt fiber (FBF). Firstly, the thermal stability, moisture absorption, and oil absorption property of these fiber additives were analyzed. Secondly, the property of SMA-13 reinforced using four types of single fibers and two kinds of composite fibers (FLF + CBF and FLF + FBF) was comprehensively analyzed. Specifically, the high-temperature performance was evaluated using the uniaxial penetration test and the rutting test, the medium-temperature anticracking property was evaluated using the IDEAL-CT test, the low-temperature property was analyzed using the beam bending test, and the water stability was studied by the freeze–thaw splitting test. Thirdly, the dynamic mechanical response of different-fibers-modified SMA-13 was evaluated using the uniaxial compression dynamic modulus test. Finally, correlation analysis between the results of dynamic modulus and the high-, medium-, and low-temperature mechanical performance was carried out. The research results reveal that the stability of CBF and FBF under thermal action is better than that of GLF and FLF, and FBF shows the best thermal stability. The oil absorption property of FLF is better than that of GLF, followed by FBF and CBF. The comprehensive mechanical properties of CBF- and FBF-reinforced SMA-13 are better than those of FLF- and GLF-modified SMA-13. CBF can better reinforce the mechanical property of SMA-13 under low and medium temperature, while FBF can better reinforce the performance of SMA-13 at high temperature. FLF/CBF- and FLF/FBF-composite-modified SMA-13 show better high-temperature mechanical performance than that of the single-fiber-reinforced mixture, and FLF has some negative impact on the properties of FLF/FBF-composite-modified SMA-13 at low temperature. Fibers have no significant influence on the water stability of the mixtures. Meanwhile, the linear correlation between the mechanical performance of all the fiber-reinforced SMA-13 and the dynamic modulus result is good.

## 1. Introduction

Asphalt pavement structure nowadays suffers from various road distresses under the comprehensive effects of human behavior and severe environmental conditions [1,2,3]. Rutting, cracks, etc., have presented road surfaces with a lot of challenges, which also increases the cost for constructing and repairing the road surfaces. Therefore, researchers around the world have focused on finding different modifiers to reinforce the properties of asphalt pavement materials [4,5].

Some researchers adopted polymer modifiers to reinforce the properties of asphalt mixtures [6,7,8]. For instance, B. Wang et al. studied the properties of asphalt mixture reinforced by antirut agents and developed a permeative antirutting agent using the Buton rock asphalt (BRA) and epoxy-based resin [9]. G. Huang et al. and S. Lv et al. evaluated the rutting and fatigue resistance of asphalt mixture reinforced by high-modulus agents [10,11]. Other researchers used fiber additives such as lignin fiber (LF), basalt fiber (BF), etc., to enhance the asphalt mixture property [12,13,14,15]. For example, Y. Hui et al. analyzed the cracking resistance of asphalt mixture modified using basalt fiber (BF), and the results indicated that BF can greatly improve the cracking resistance [16]. D. Ren et al. adopted some surface sizing agents to modify BF and studied the performance of an asphalt mixture prepared using the modified BFs [17,18]. Tamrin et al. studied the property of an asphalt mixture reinforced using the composite modifier of lignin fiber and an organic modifier [19].

Among all the additives, fiber additives are more and more popular because they are eco-friendly and the raw materials are easily found on Earth [20,21,22]. References indicate that LF (lignin fiber) and BF (basalt fiber) are two types of plant and mineral fiber being used in asphalt mixture [23,24]. Therefore, this paper chooses these to analyze their impact on asphalt mixtures. Regarding the mixture type, this paper focuses on the stone matrix asphalt with the nominal maximum aggregate size of 13 mm (SMA-13), for the reason that it is one of the most widely used mixture types for the upper surface [25].

However, apart from the traditional flocculent LF (lignin fiber) and short-cut BF (basalt fiber), two new forms of LF and BF, namely, granular LF (GLF) and flocculent BF (FBF), have emerged. The differences between FBF/FLF and BF/LF are the morphology. The research related to the effect of the morphology of fiber on the fiber-reinforced asphalt concrete or mixture material is limited. Thus, it is worth analyzing its effect on the fiber-reinforced mixture. Furthermore, the hybrid use of fiber additives with different morphology is also worthy of exploration.

Therefore, this paper focuses on analyzing the effect of flocculent lignin fiber (FLF), granular lignin fiber (GLF), chopped basalt fiber (CBF), and flocculent basalt fiber (FBF) on the high-temperature antirutting performance, low- and medium-temperature crack resistance, water stability, and dynamic mechanical response of SMA-13. Meanwhile, since the flocculent lignin fiber (FLF) is suggested for use in SMA-13 to absorb the oil in the literature, the combined use of FLF and CBF or FBF in SMA-13 mixture is analyzed. This paper could help to guide the scientific selection of fiber additives in the design and application of SMA-13 mixtures.

## 2. Materials and Methods

### 2.1. Materials

#### 2.1.1. Raw Materials

**Figure 1:**
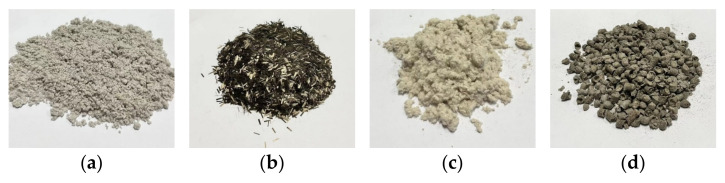
Morphologies of fiber additives. (**a**) Flocculent basalt fiber; (**b**) chopped basalt fiber; (**c**) flocculent lignin fiber; (**d**) granular lignin fiber.

The coarse aggregate used in the current study was grid from basalt rock, and the limestone fine aggregate was selected. Filler powder was adopted as limestone powder, with the properties shown in Table 1. SBS-modified asphalt with the grade of PG76-22 was used, and the properties are shown in Table 2. Four type of fibers, flocculent basalt fiber (FBF), chopped basalt fiber (CBF), flocculent lignin fiber (FLF), and granular lignin fiber (GLF), were selected, and the fiber properties are shown in Table 3. Macroscopic morphologies of the different fiber modifiers are shown in Figure 1.

#### 2.1.2. Gradation Design

**Figure 2:**
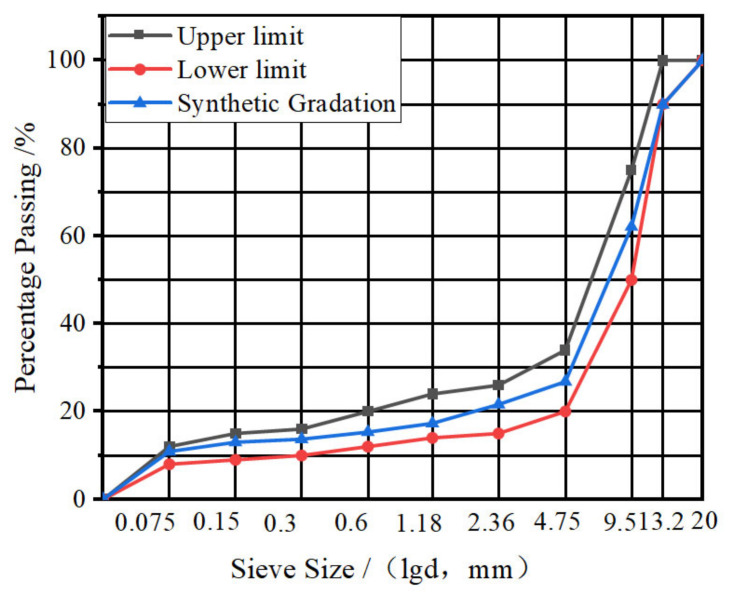
Gradation design curve of SMA-13 mixture.

The gradation type of the mixture is determined as SMA-13 (stone matrix asphalt whose nominal maximum aggregate size is 13.2 mm). The gradation curve is shown in Figure 2. The upper and lower limits of the gradation curve are obtained from the specification of JTG F40 2004 [26]. The final adopted gradation curve of the SMA-13 asphalt mixture in this study is called the synthetic gradation curve, because it is obtained by the combination of several different kinds of stones with different size ranges. The adopted fiber addition design, the corresponding optimum asphalt content, and the volumetric parameters are shown in Table 4. The Cantabro test result and the Schellenberg binder drainage test results are shown in Table 5. Generally, there are six types of fiber addition schemes, including four types of single fiber and two types of composite fiber. Specifically, the SMA-13 with the flocculent lignin fiber (FLF) is regarded as the control mixture, because FLF is required to be added by the specification of JTG F40 2004 and many references [27].

### 2.2. Experiments

#### 2.2.1. High-Temperature Property Test Method

The high-temperature rutting test was adopted for high-temperature performance analysis according to JTG E20-2011 [28]. As is indicated in the regulation, the specimen is a cuboid with the size of 300 × 300 × 50 mm (length × width × height). The temperature during the experiment was controlled at 60 °C, while 0.7 MPa was set as the test wheel pressure. The mathematical calculating method of dynamic stability is illustrated in Equation (1).

(1) $$DS=\frac{(t_{2}-t_{1})\times N}{d_{2}-d_{1}}\times C_{1}\times C_{2}$$

where *DS* is index of dynamic stability of the specimen; *d_1_* represents the rutting deformation at forty-five minutes; *d_2_* corresponds to the sixty-minutes deformation; *C_1_* and *C_2_* are the testing parameters and their value is set as 1; *N* is the rolling speed of the testing wheel and is normally determined as 42 times per minute.

#### 2.2.2. Low-Temperature Property Test

The low-temperature performance test was conducted by trabecular test following JTG E20-2011, in which trabecular specimens are prismatic beam with length × width × height of 250 mm × 30 mm × 35 mm, respectively. The experiment was conducted using a UTM-25 testing machine, with the temperature maintained at −10 °C and the pressure velocity of the specimens maintained at 50 mm/min. The UTM-25 testing machine is made by IPC Global Co., Ltd., Melbourne, Australia. The calculation formulas for flexural tensile strength and maximum flexural strain are shown in Equations (2) and (3).

(2) $$R_{B}=\frac{3LP_{B}}{2bh^{2}}$$

(3) $$\varepsilon_{B}=\frac{6hd}{L^{2}}$$

where *b* and *h* are the width and height of the specimen; *L* is the test span of the sample in the test; *P_B_* is the peak load when the specimen breaks; *d* is the deflection at mid-span when the sample reaches failure.

#### 2.2.3. Medium-Temperature Performance Test

The medium-temperature property was evaluated by the IDEAL-CT test [29,30,31]. The cylinder-shaped specimens were prepared by using the rotatory-compaction machine, and the diameter and thickness of the test sample were 150 mm and 62 mm. The experiment was carried out by UTM-25 testing machine, and the displacement loading rate and experiment temperature was set as 50 mm/min and 25 °C. The evaluation indexes are listed in Equations (4) and (5).

(4) $$CT_{\mathrm{Index}}=\frac{G_{f}}{\left|m_{75}\right|}\times (\frac{l_{75}}{D})$$

(5) $$\left|m_{75}\right|=\left|\left(p_{85}-p_{65}\right)/\left(l_{85}-l_{65}\right)\right|$$

where *CT*_Index_ represents the cracking propagation property; *G*_f_ is the cracking energy during the experimentation process; $\left|m_{75}\right|$ is the slope value of the post peak curve when the force is 75% of the maximum force, and *l*_75_ is the corresponding displacement at this post peak curve point; *D* is 150 mm and refers to the diameter of the test sample.

#### 2.2.4. Water Stability Test

The water stability was tested by the freeze–thaw splitting test (FTST) following the instruction listed in JTG E20-2011. The calculation formulas of the test indexes are shown in Equations (6)–(8).

(6) $$R_{T1}=0.006287P_{T1}\times h_{1}$$

(7) $$\begin{array}{c} R_{T2}=0.006287P_{T2}\times h_{2} \end{array}$$

(8) $$TSR=\frac{R_{T2}}{R_{T1}}\times 100$$

where *R_T_*_1_ represents the splitting tensile strength (STS) of the test samples which are not under the freeze–thaw actions, and this is regarded as the test group 1; *R_T_*_2_ represents the STS of the second test groups, in which the samples have been through the freeze–thaw process. *P_T_*_1_ and *P_T_*_2_ are the tensile force of the first and second test group. *h*_1_ and *h*_2_ are the specimen height. *TSR* is the ratio between the average STS value of the second (*R_T_*_2_) and the first (*R_T_*_1_) specimen group.

#### 2.2.5. Dynamic Modulus Test Method

The compression dynamic modulus test (CDM) was adopted to evaluate the dynamic response property of the mixture, and standard test specimens with height of 150 mm and diameter of 100 mm were used. A multifunctional instrument was selected to apply sine wave load to the top of the specimen. The test temperature was selected as −10, 5 °C, 20 °C, 35 °C, and 50 °C, and six loading frequencies were selected for each temperature (25 Hz, 10 Hz, 5 Hz, 1 Hz, 0.5 Hz, and 0.1 Hz). The experiment started from low to high temperature, and was conducted from high to low frequency at the same temperature. Then, a mathematical model was adopted to fit the master curve of dynamic modulus. The calculation formulas are shown in Equations (9)–(11).

(9) $$\begin{array}{c} \log\left(\left|E^{*}\right|\right)=\delta +\frac{\alpha -\delta }{e^{\beta +\gamma \log t_{r}}} \end{array}$$

(10) $$\begin{array}{c} t_{r}=\frac{t}{\alpha_{T}} \end{array}$$

(11) $$\begin{array}{c} \log\left(t_{r}\right)=\log\left(t\right)-\log\left(\alpha_{T}\right) \end{array}$$

where $\left|E^{*}\right|$ is the dynamic modulus in the main master curve; *t_r_* is the master curve frequency at the reference temperature; δ and α are the parameters of the model shown in Equation (11); β, γ are the descriptions parameters for the shape of the master curve; *t* is the action frequency of the load at the converted temperature; $\alpha_{T}$ is the shift factor.

## 3. Results and Discussion

### 3.1. High-Temperature Rutting Test Results

**Figure 3:**
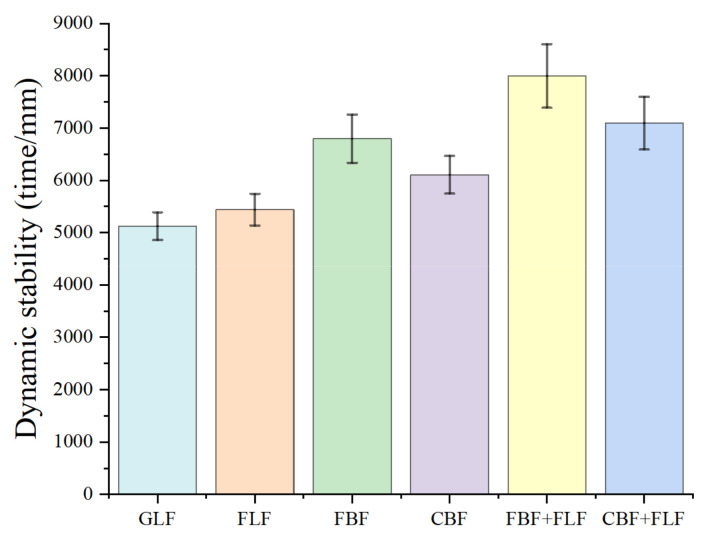
The results of rutting test at high temperature.

The dynamic stability (DS) results of the rutting test are illustrated in Figure 3. It can be seen from Figure 3 that the DS values of the mixtures with BF are better than those of mixtures with LF and the improvement range is between 12.3% to 32.7%, indicating that BFs have better reinforcing effect on the high-temperature dynamic stability than LFs. In addition, the dynamic stability of SMA-13 with flocculent BF (FBF) is better than the mixture with chopped BF (CBF), and the DS of the mixture with FBF increases by 11.3% compared to the mixture with granular lignin fiber (GLF). Similar differences can be found between the mixtures with flocculent lignin fiber (FLF) and GLF. This indicates that fiber morphologies also have an influence on the anti-high-temperature property, and flocculent fibers have better reinforcing effect on the high-temperature dynamic stability. Furthermore, DS values of the mixture with composite fibers are much higher than those of mixtures with single fiber, despite the fiber type, and the composite of FBF + FLF-reinforced mixture presents the best high-temperature rutting resistance.

### 3.2. Low-Temperature Trabecular Bending Test Results

**Figure 4:**
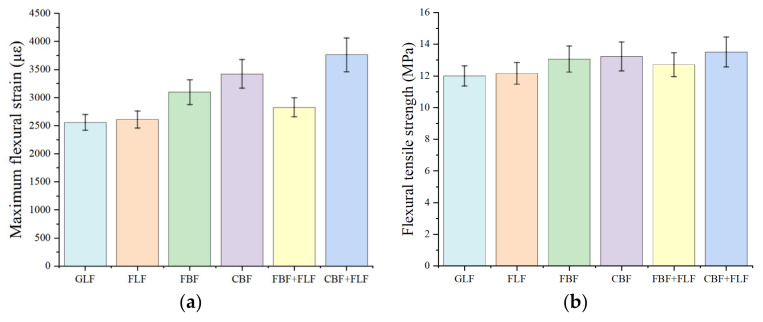
Low-temperature trabecular bending test results: (**a**) Maximum bending tensile strain. (**b**) Flexural tensile strength.

The maximum flexural strain and flexural tensile strength results of the low-temperature test are shown in Figure 4. It can be observed from Figure 4 that maximum flexural strain of SMA-13 mixtures with BFs are over 3000 με, which can be used for the extreme cold regions with temperature as low as −37 °C. Meanwhile, the maximum flexural strain of mixtures with LFs are around 2500 με, which can be used for cold regions with temperatures as low as −37 °C to −21.5 °C, according to the JTG-F40-2004. This means that BF has a better reinforcing effect on the low-temperature resistance than LF. These findings are consistent with the results from reference [32]. In addition, the maximum flexural strain of the asphalt mixture with CBF is 10.5% higher than that of asphalt mixture with FBF, while the flexural strain of mixtures modified by LFs with different morphologies is almost the same. Furthermore, it is found that CBF has a better reinforcing effect on the maximum flexural strain of FLF-modified SMA-13 than FBF. Meanwhile, FLF could decrease the maximum flexural strain of the FBF-modified mixture and increase the maximum flexural strain of the CBF-modified mixture. This phenomenon is mainly because CBF could form a solid three-dimensional network structure in the mixtures, and FLF could absorb the redundant oil in the mixture [33,34]. These two fibers could have synergic effects on the properties of the mixture in the low-temperature cracking failure process of the mixture. However, when FLF and FBF are used together, the network structure formed by these two flocculent fibers might not be good as the FLF/CBF-modified mixture, because the two types of flocculent fibers might agglomerate with each other, resulting in some stress concentration at low temperatures [35].

### 3.3. Medium-Temperature IDEAL-CT Test Results

**Figure 5:**
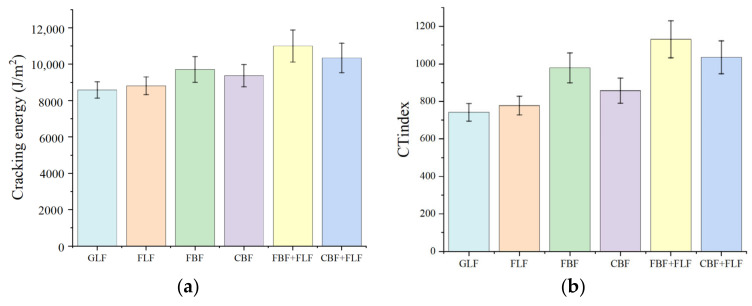
The results of the IDEAL-CT test: (**a**) Cracking energy. (**b**) Crack resistance index.

The cracking energy and *CT*_Index_ results of the IDEAL-CT test are illustrated in Figure 5. The cracking energy and *CT*_Index_ calculated from the IDEAL-CT load–displacement curve indicate that the cracking resistance and the anticracking propagation ability of SMA-13 with BFs is better than those with LFs. The *CT*_Index_ of the mixture with BFs improves by 9.2% to 24.2% compared to the mixture with LFs. The cracking resistance and anticracking propagation ability of the samples with composite fiber are better than those with single fibers. The modification effect of flocculent LF (FLF) is better than granular lignin fiber (GLF), and the reinforcing degree of flocculent BF (FBF) is better than the chopped BF (CBF). The *CT*_Index_ of mixture with FBF increases by 14.2% compared to the mixture with CBF. This pattern is also observed in the composite-fiber-modified SMA-13. Flocculent BF (FBF) can better increase the anticracking ability of the traditional SMA-13 with flocculent LF (FLF) than the chopped BF (CBF).

### 3.4. Freeze–Thaw Splitting Test Results

**Figure 6:**
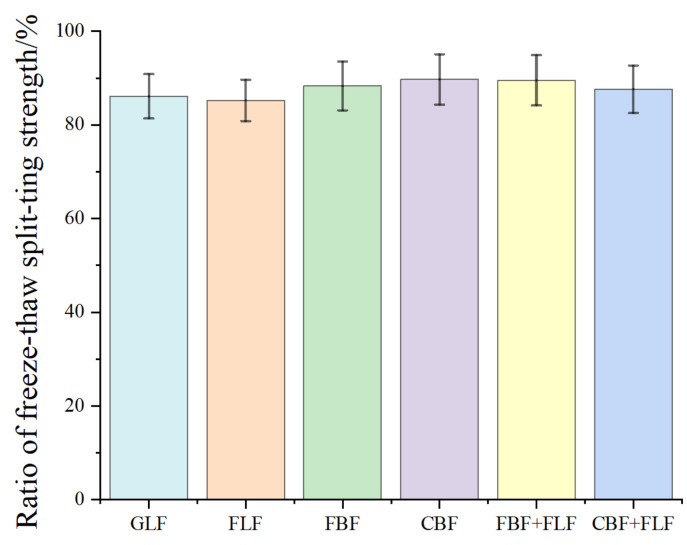
The results of the water stability test.

The TSR results of the freeze–thaw splitting test are illustrated in Figure 6. It can be seen from Figure 6 that the TSR values of all the six type of mixtures are within the range of 85–89%, which indicates that the six types of mixtures all meet the minimum limitation value of 80% according to JTG E20-2011. This means that these fibers have no significant influence on the water stability of the mixtures.

### 3.5. Dynamic Modulus Test Results

**Figure 7:**
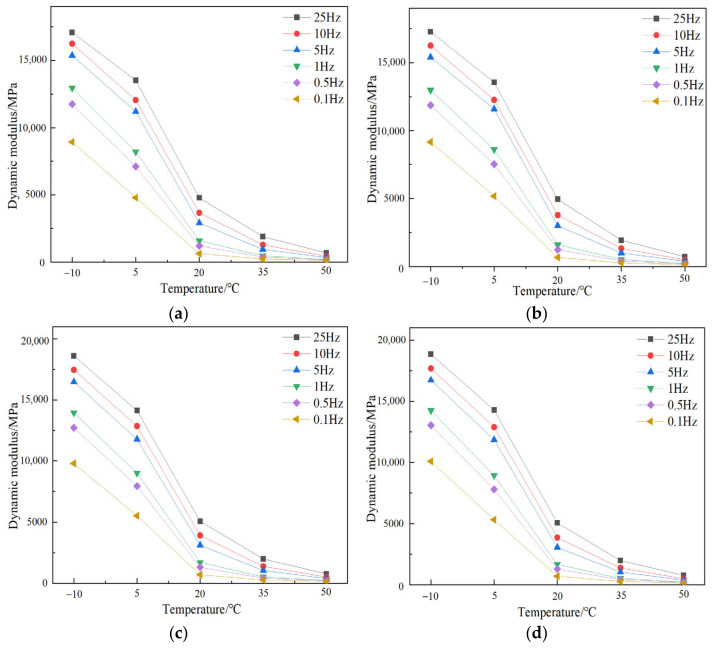

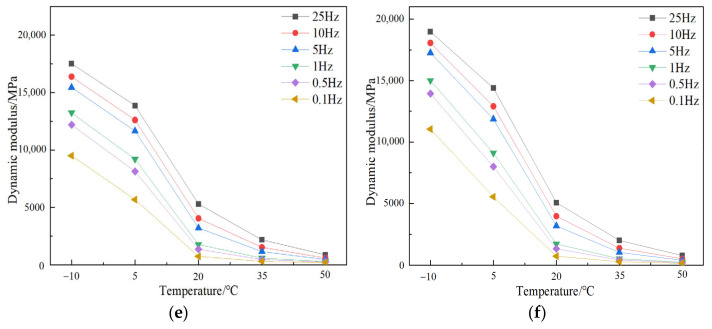
Dynamic modulus of different-fiber-modified SMA-13 under various temperatures: (**a**) GLF, (**b**) FLF, (**c**) FBF, (**d**) CBF, (**e**) FBF + FLF, and (**f**) CBF + FLF.

The asphalt mixture is mainly elastic at low temperature and viscous at high temperature, which is primarily due to the complexity of the asphalt [36]. Figure 7 shows the variation of dynamic modulus of different mixtures with temperature. As shown in Figure 7, the DM values of the mixture with all types of fibers decrease with the increase in temperature. Meanwhile, the changing trend is similar and consistent for all test samples. When the temperature is below 20 °C, the dynamic modulus decreases sharply with the increase in temperature; at 20 °C, there is a significant inflection point. When the temperature exceeds 20 °C, the decreasing rate of the dynamic modulus tends to flatten out; when the temperature rises to 50 °C, the dynamic modulus at each frequency tends to be close to each other. These findings indicate that the DM values vary more obviously at low temperatures.

**Figure 8:**
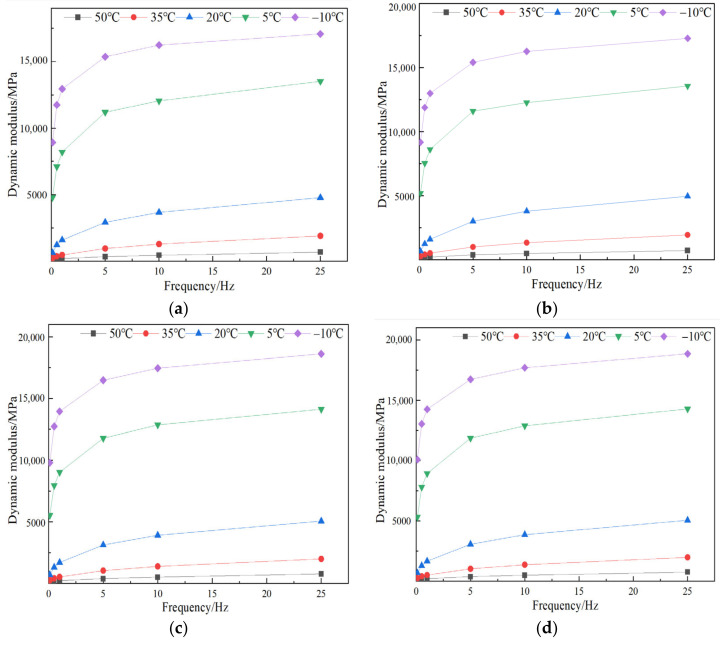

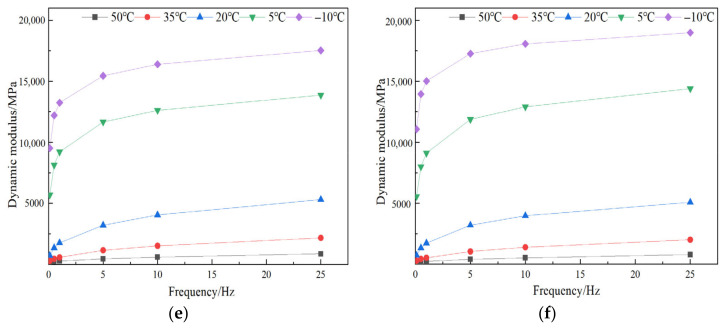
Dynamic modulus of different-fiber-modified SMA-13 under different frequencies: (**a**) GLF, (**b**) FLF, (**c**) FBF, (**d**) CBF, (**e**) FBF + FLF, and (**f**) CBF + FLF.

Figure 8 shows the variation of dynamic modulus with frequency. From Figure 8, it can be seen that DM increases with the frequency at the same temperature. Meanwhile, the DM value at low temperature varies more greatly than the DM value at high temperature when the frequency varies from 0.1 to 25 Hz. The changing degree of DM value is the smallest when the frequency is small and the temperature is high.

**Figure 9:**
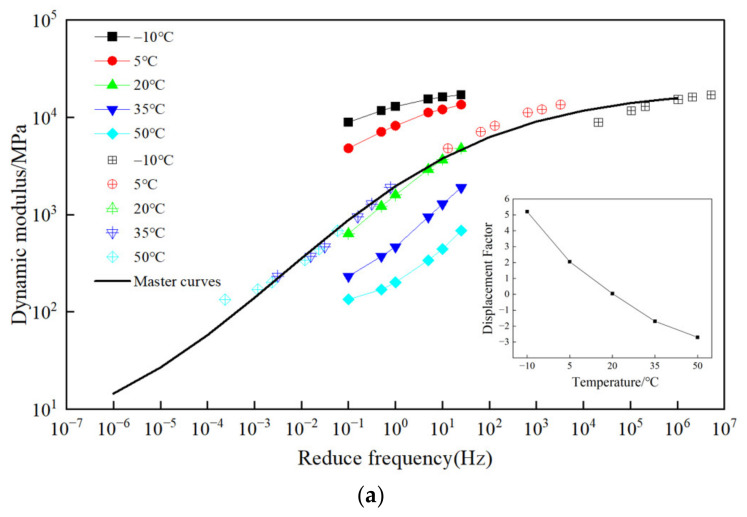

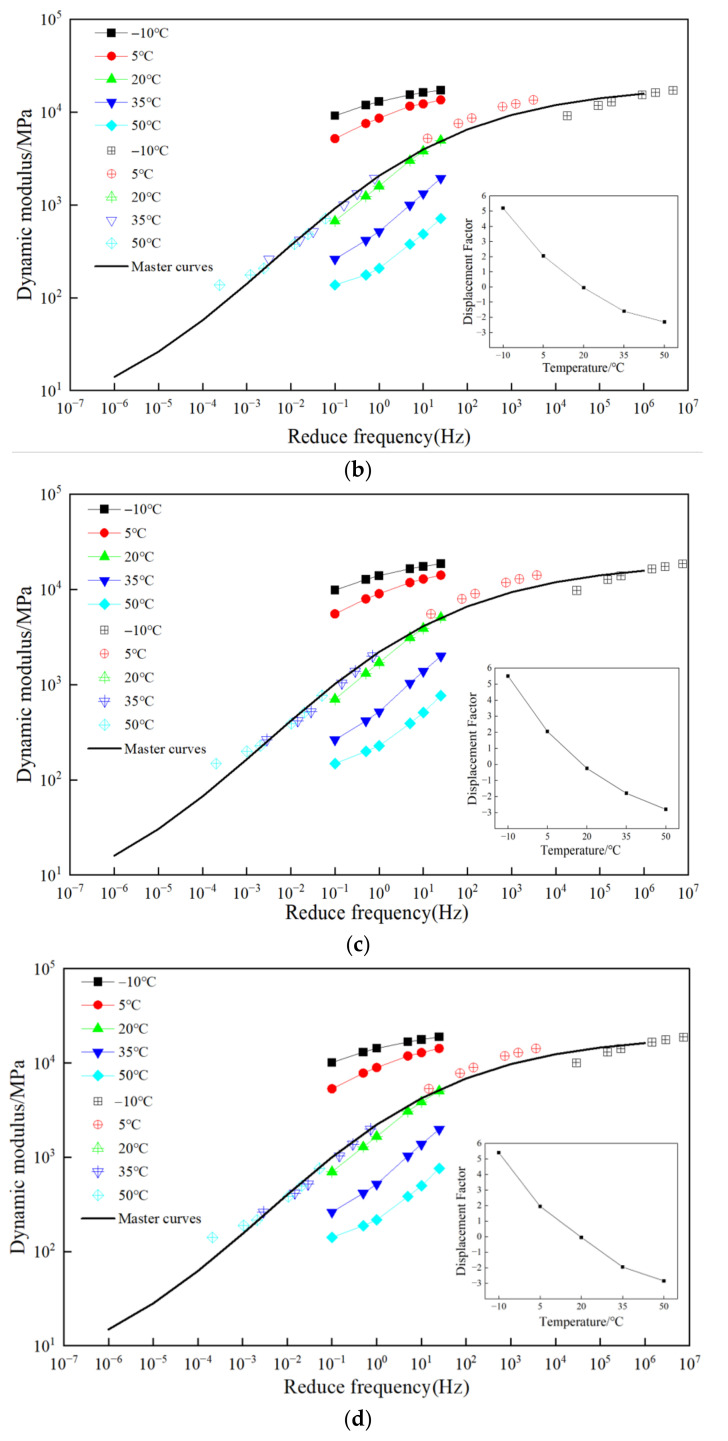

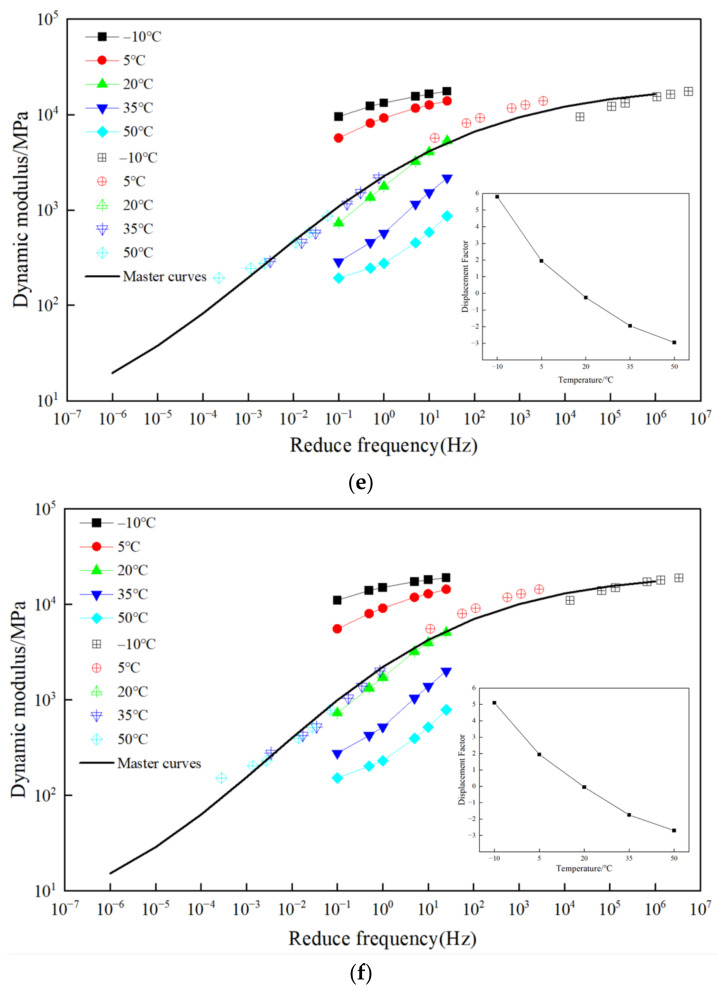
Master curve of different types of fiber asphalt mixture: (**a**) GLF, (**b**) FLF, (**c**) FBF, (**d**) CBF, (**e**) FBF + FLF, and (**f**) CBF + FLF.

From Figure 9a–f, it can be seen that there is a functional relationship between shift factor and temperature, and the variation range of shift factor can reflect the impact degree of temperature on the dynamic mechanical behavior of the mixtures. It reveals that the shift factor decreases with the increase in temperature. The master curve can extend the test frequency of 0.01~25 Hz to 10^−6^~10^6^ Hz. It breaks through the limitation of test equipment and has a wider frequency domain and a higher DM value. The DM value of the fiber-modified mixture is positively correlated with the loading frequency, in which the increasing trend is gentle at the high frequency; an S-shaped curve is observed for all the samples.

**Figure 10:**
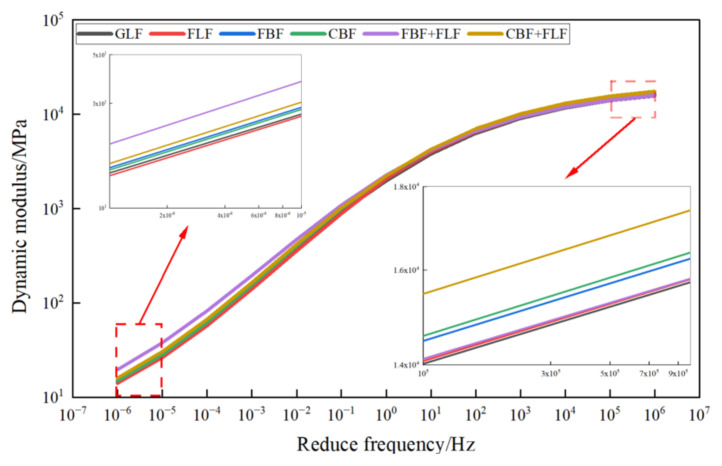
Total master curve.

However, Figure 9a–f cannot well show the influence of various fiber additives on the dynamic modulus value. To further analyze the specific effect of fiber types on the dynamic mechanical response of the mixture samples, all the master curves of the test specimens at 20 °C are illustrated in Figure 10.

From Figure 10 it can be seen that in the low-frequency (corresponding to the high-temperature) area, the difference of DM values of different fiber SMA-13s is obvious, indicating that the fiber type and fiber morphology had a greater influence under these conditions. In this area, the DM value of SMA-13 with composite fibers is greater than the mixture with single fibers. BFs had better enhancing effect on the DM value than LFs. The FBF + FLF mixture had the largest DM value at low frequencies. This indicates that it has the highest temperature resistance performance, which is consistent with the results of the rutting experiment.

Meanwhile, the effect of different fibers on the DM values of different fiber SMA-13 shows different trends in the high-frequency (corresponding to low-temperature) area. The CBF + FLF-modified mixture has the biggest DM value at high frequencies, and the FBF + FLF mixture has a smaller DM value than the BF-modified mixture. This is also in accordance with the low-temperature maximum bending tensile strain and flexural tensile strength results introduced before.

**Figure 11:**
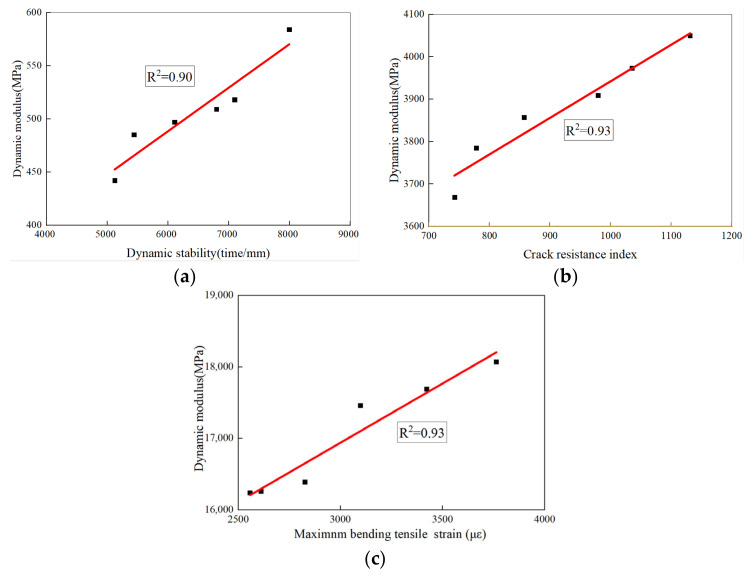
Correlation analysis: (**a**) Correlation analysis between dynamic stability and dynamic modulus; (**b**) correlation analysis between crack resistance index and dynamic modulus; (**c**) correlation analysis between maximum bending strain and dynamic modulus.

In order to analyze the correlation between the dynamic mechanical behavior and the various road performances of the SMA-13s studied before, the measured dynamic moduli at 50 °C, 20 °C, and −10 °C under 10 Hz were adopted to conduct a correlation analysis between the high-temperature dynamic stability, medium-temperature anticrack ability, and low-temperature property of the asphalt mixtures. As shown in Figure 11a–c, the correlation coefficients reach 0.90, 0.93, and 0.93, respectively, indicating good consistency between the dynamic modulus and road performance of fiber asphalt mixtures.

## 4. Conclusions

In this study, different types of fibers (GLF, FLF, CBF, FBF, FBF + FLF, CBF + FLF) were selected to prepare SMA asphalt mixtures. The influence of various fibers on the high-temperature antirutting performance, low- and medium-temperature crack resistance, water stability, dynamic mechanical response, etc., were analyzed. The following conclusions are drawn:

(1) BFs have a better reinforcing effect on the high-temperature dynamic stability and medium-temperature cracking resistance than LFs. Flocculent fibers have a better reinforcing effect on the high-temperature dynamic stability and medium-temperature cracking resistance than the fibers with other morphologies. The FBF + FLF-reinforced mixture presents the best high-temperature rutting resistance and medium-temperature cracking resistance.

(2) BF has a better reinforcing effect on the low-temperature resistance than LF. The maximum flexural strain of the asphalt mixture with CBF is 10.5% higher than that of the asphalt mixture with FBF, while the flexural strain of mixtures modified by LFs with different morphologies is almost the same. CBF has better reinforcing effect on the low-temperature maximum flexural strain of FLF-modified SMA-13 than FBF. FLF could decrease the maximum flexural strain of the FBF-modified mixture and increase the maximum flexural strain of the CBF-modified mixture.

(3) The TSR values of all the six type of mixtures meet the minimum limitation value of 80% according to the specification. This means that these fibers have no significant influence on the water stability of the mixtures.

(4) The DM value is negatively correlated with the test temperature and positively correlated with frequency. The changing degree of DM value is the smallest when the frequency is small and temperature is high. Shift factor decreases with the increase in temperature.

(5) According to the master curve of DM of each fiber SMA-13, it can be inferred that the FBF + FLF mixture has the largest DM value at low frequencies and the CBF + FLF mixture has the biggest modulus at high frequencies. The differences between the DM value of different SMA-13 with various fibers is smaller when the frequency increases.

(6) The correlation between the dynamic modulus and the high-temperature dynamic stability, medium-temperature cracking resistance, and low-temperature property of fiber asphalt mixtures is good, and the R^2^ values are all over 0.9. This indicates that the dynamic modulus values can be used to reveal the road performance of mixture to some extent.

Overall, the scope of this study aimed at analyzing the effect of fiber type, fiber morphology, fiber combination, etc., on the performance of the asphalt mixture. The findings could be regarded as references for the design of fiber-reinforced asphalt mixtures.

## Figures and Tables

**Table 1:** Indexes of mineral powder properties.

Index		Result
Moisture content/%		0.8
Relative density		2.702
Hydrophilic coefficient		0.8
Particle size range	<0.6 mm	100
	<0.15 mm	99.0
	<0.075 mm	88.5

**Table 2:** Indexes of asphalt performance.

Index		Result
Needle penetration (25 °C)/0.1 mm		56
Softening point/°C		81
Ductility (5 cm/min, 5 °C)/cm		48
Elastic recovery (25 °C)/%		96
Residue after RTFOT	Mass change/%	−0.1
	Penetration ratio/%	86
	5 °C Residual ductility/cm	37

**Table 3:** Indexes of different types of fibers.

Index	FLF	GLF	CBF	FBF
Diameter/μm	≈13	4.1	16	5
Length/mm	0.8	3~6	6	3~6
Density/g·cm^−3^	0.910	1.112	2.712	2.711
Tensile strength/MPa	<300	-	≥2000	-
Oil absorption rate	4.3	6.5	1.2	2.3
Hygroscopicity/%	12.7	30.3	2.7	5.8

**Table 4:** Fiber blending scheme and the mixture gradation design parameters.

Mixture Gradation	Fiber Type	Fiber Content/%	Optimum Asphalt Content/%	Void Ratio VV/%	Voids of Mineral Aggregate VMA/%	Voids Filled with Asphalt VFA/%
SMA-13	GLF	0.4	5.46	3.8	17.1	78.0
	FLF	0.3	5.64	3.5	17.3	79.5
	FBF	0.4	5.46	3.6	16.5	78.2
	CBF	0.4	5.37	3.6	16.6	77.2
	FBF + FLF	0.3 + 0.1	5.55	3.2	17.2	80.3
	CBF + FLF	0.3 + 0.1	5.46	3.2	17.4	81.0

**Table 5:** Cantabro scattering and Schellenberg binder drainage test results.

Type	Asphalt Scattering Loss Δ*S*/%	Asphalt Drainage Loss Δm/%
GLF	5.4	0.07
FLF	5.3	0.08
FBF	5.2	0.11
CBF	5.1	0.12
FBF + FLF	5.4	0.09
CBF + FLF	5.5	0.07

## Data Availability

The data presented in this study are available on request from the corresponding author.
